# Enhanced electrochemical performance of eco-friendly Cr-doped ZnO/RGO nanocomposites for pioneering supercapacitor applications

**DOI:** 10.3389/fchem.2026.1830818

**Published:** 2026-06-11

**Authors:** Faiqa Arshad, Sammia Shahid, Sana Mansoor, Momna Qayyum, Mohsin Javed, Ayesha Khadim, Syed Kashif Ali, Ali Bahadur, Shahid Iqbal, Sajid Mahmood, Meznah M. Alanazi, Shaimaa A. M. Abdelmohsen

**Affiliations:** 1 Department of Chemistry, School of Science, University of Management and Technology, Lahore, Pakistan; 2 Department of Physical Sciences, Chemistry Division, College of Science, Jazan University, Jazan, Saudi Arabia; 3 Engineering and Technology Research Center, Jazan University, Jazan, Saudi Arabia; 4 Nanomaterials Research Center, Department of Chemistry, College of Science, Mathematics, and Technology, Wenzhou-Kean University, Wenzhou, Zhejiang, China; 5 Dorothy and George Hennings College of Science, Mathematics and Technology, Kean University, Union, NJ, United States; 6 Nottingham Ningbo China Beacons of Excellence Research and Innovation Institute, University of Nottingham Ningbo China, Ningbo, China; 7 Low Dimensional Materials Research Center at Khazar University, Baku, Azerbaijan; 8 Department of Physics, College of Science, Princess Nourah bint Abdulrahman University, Riyadh, Saudi Arabia

**Keywords:** co-precipitation, Cr-doping, Cr-ZnO/RGO, electrochemical analysis, supercapacitors, ZnO

## Abstract

Herein, a straightforward, and mild synthetic co-precipitation method is used to prepare Cr-ZnO/RGO nanocomposite to be used in supercapacitor applications. First, the chromium (Cr) doping concentration in zinc oxide (ZnO) nanoparticles was systematically varied (2, 4, 6, 8, and 10%) to evaluate its influence on the electrochemical performance. The composite was constructed by combining reduced graphene oxide (RGO) with 8% Cr-ZnO, which showed the best redox potential among the compositions under investigation. The structural, morphological, compositional, and optical properties were characterized using the complementary analytical techniques. Cyclic voltammetry (CV) and EIS were used to assess electrochemical performance in 1M KOH. The optimized 8% Cr-doped ZnO/RGO nanocomposite exhibited a band gap of 3.59 eV. Electrochemical evaluation revealed a high specific capacitance of 703 F g^−1^ at a scan rate of 100 mVs^-1^. The CV profiles were quasi-rectangular in nature, indicating a combination of electric double-layer and pseudocapacitive charge storage. Furthermore, the electrode demonstrated 89% capacitance retention over 5,000 charge-discharge cycles with 99% Coulombic efficiency, confirming stable electrochemical behavior. EIS analysis revealed low internal resistance and enhanced charge transport with considerably reduced surface resistance (Ru) and solution resistance (Rp) values. The synergistic effect between the optimal chromium doping and integration with RGO was attributed to this improved efficiency. These results demonstrate that Cr-ZnO/RGO nanocomposites can be used as a promising electrode material in supercapacitor applications.

## 1 Introduction

Growing populations, developing global economy, and greater reliance on energy-intensive machinery have all contributed to rising global energy consumption and carbon dioxide emissions over past few decades (Kandhasamy et al., 2024). As a result, the need for ecologically friendly energy storage devices (ESDs) have increased dramatically. A number of sustainable energy storage technologies are fulfilling this requirement. Electrochemical energy storage devices (EESDs) have attracted significant interest among them due to their clean, safe, reliable, and efficient energy storage technology. The three of the main kinds of electrochemical energy storage devices includes batteries, capacitors, and supercapacitors, each depending on different energy storage processes (Ren M. et al., 2021). In the recent years, energy storage devices have become increasingly necessary for consumer and industrial power control systems due to the advancements in technology and automation (Abdul Bashid et al., 2017). To fulfill these requirements, it is very essential to develop the energy storage devices that are flexible, lightweight, have fast charging and discharging along with the robust life cycles, with wide operating range, are high-energy-density, and are also environmentally friendly. The supercapacitors fit to this description well (Chaudhary et al., 2019).

Because of their higher specific capacitance and favorable temperature characteristics than the batteries, supercapacitors (SCs) have attracted a lot of the attention (Kant et al., 2022). They serves as intermediaries in energy storage for use in industrial equipment, transportation, electrically powered automobiles, and portable devices, functioning alongside fuel cells, power sources (Joshi et al., 2025; Sharma and Kumar, 2020). The performance of SCs are primarily influenced by type of the material used for the electrodes and their accessible potential window. Additionally, the charge storage mechanism plays a crucial role in determining the overall electrochemical behavior (Ramachandran et al., 2025a). It is well established that electrochemical energy storage is governed by a complex interplay of physical and chemical processes, specifically electric double layer capacitance (EDLC), pseudocapacitance and diffusion-controlled faradic responses (Ramachandran et al., 2025b). Depending on the relative contribution of these mechanism, electrode material may exhibit behavior ranging from ideal capacitive to battery-type characteristics (Zan et al., 2024). Therefore, distinguishing between surface-controlled and diffusion controlled processes is very essential for accurately interpreting the charge storage mechanism and electrochemical performance (Pandey et al., 2026).

Research continues to focus on improving of the materials for optimal supercapacitors (SCs) (Miller et al., 2018). Most recent researches has been directed toward the creation of electrode materials with exceptionally high specific energy and specific power (Ren K. et al., 2021). MOFs have been regarded as potentially useful supercapacitor electrode materials. But their poor cycle stability, low electrical conductivity, aggregation propensity, and low capacity have made it necessary to investigate better design approaches (Muthu et al., 2025). Carbon-based materials outperform conventional metal electrodes, owing to their large pore size, loose structural organization, uneven density, massive grain size, and the significant anisotropy (Jin et al., 2017; Bashir et al., 2025). Excellent conductivity and light density are achieved by using carbon nanomaterials like the graphene, carbon nanotubes, and graphite oxides (Kolahdouz et al., 2022). The nonmagnetic properties of such materials also restrict their usage despite their excellent dielectric properties (Singh et al., 2019). In order to enhance their compatibility with carbon-based nanomaterials, the metal oxides have been investigated. By modifying the atomic structure of the graphene, metal oxides can enhance its mechanical, chemical, and physical properties, which can allow for the better synergistic interaction for the range of applications (Sun et al., 2016). In this context, transition metal oxides (TMO) offer a novel approach to the development of energy storage materials due to their excellent stability, high capacity, good cycle performance, and affordability (Wei et al., 2022).

Of all the transition metal oxide semiconductors, zinc oxide (ZnO) has received considerable interest in the scientific community and industry because of its high oxidation potential, favorable optoelectronic and catalytic characteristics, non-toxicity, chemical stability, and the affordability (Kareem et al., 2022). ZnO NPs are n-type semiconductors that falls under group II-VI (Adedokun et al., 2020) that have numerous applications, such as the light-emitting diodes, (Voss and Waldvogel, 2017), gas sensors (Al-darwesh et al., 2023), nano-power generators (Le et al., 2020), and the detection of ultraviolet (UV) radiations (Choi et al., 2020). Other material characteristics of ZnO are that it has good electrical conductivity (Liu et al., 2021), excellent chemical stability (Jiang et al., 2023), and wide band gap (Rodwihok et al., 2019). Moreover, the ZnO has piezoelectric properties and self-carrier generation when stressed or twisted. ZnO based composites that have a high dielectric constant and a low dielectric loss can also find application in semiconductor electronics like the memory devices, integrated circuits (ICs), multilayer capacitors, as well as the supercapacitors (Wei et al., 2016).

The manipulation of the structural, photosensitive, electrical, and magnetic features of the ZnO NPs are possible through defect engineering via the control of their types and densities (Worku et al., 2021). This gives rise to diversity in sizes and the morphological variations. Doping is a viable method of purposely incorporating impurities into the nanomaterials to alter their distinctive properties (Worku et al., 2021). ZnO NPs could be doped with numerous materials, which leads to phenomenal changes in their bandgap, optical characteristics, electrocatalytic activity (Wang et al., 2020) and electronic configuration (Muktaridha et al., 2021). Sb, Ag, Cr, Fe, Mn, Co, P, In, Ga, Li, Al, As, Mg, and Sn are few of the examples of transition metals that can be used for doping to control their size and morphology (Hessien et al., 2021). Among them Cr ions have attracted a lot of interest as dopant, because their Cr^3+^ radius is closer to Zn^2+^, allowing Cr^3+^ to effectively substitute for the Zn^2+^ site in the ZnO crystal lattice (Carofiglio et al., 2020). The band gap energy and the dielectric characteristics of ZnO host nanomaterials can also be altered by the dopant chromium, which increase charge carrier density in comparison to that of the undoped ZnO. According to earlier research, Cr doping has significant impact on ZnO’s magnetic, optical, and dielectric characteristics. The doping of Cr in Zn sites reduces grain size, contributing to nanoscale materials that exhibit unique chemical and physical properties, along with significant surface areas and quantum confinement effects. The hexagonal close-packed lattice of ZnO allows for Cr incorporation, which can modify morphology for device applications and leads to an increased energy band gap, indicating the quantum confinement effect (Mangamma et al., 2021).

Changes in the percentage of Cr ions cause adjustments in the carrier concentration, microstrain, and native defects into the ZnO lattice framework (Debnath et al., 2018). Recent studies, such as those by Adamopoulous et al., has documented the enhanced electrical characteristics of Ag-doped ZnO-based NCs following the integration of reduced graphene (Adamopoulos et al., 2023). Debnath et al. reported the synthesis of Cr-doped ZnO nanomaterial via a hydrothermal and co-precipitation method, demonstrating that the synthesis methods for ZnO nanomaterials greatly influence their dielectric properties. They observed that hydrothermal method produces rod-like structures with larger grain sizes and greater structural asymmetry, leading to higher dielectric permittivity due to increased polarization effects under an electric field. In contrast, the co-precipitation method yields more symmetric, spherical grain structures with smaller sizes, where the grain boundary effect is lower, resulting in reduced electron tunneling. The findings underscore the crucial role of synthesis procedures in enhancing the charge transport properties of ZnO nanomaterials (Debnath et al., 2022). Chen et al. reported a MOF-based metal oxide heterostructure (ZnO/NiO-350) with a flower-like morphology. With a specific capacitance of 543 F g^−1^ at 1 A g^−1^ and an 80% retention capacity over 5,000 cycles, the ZnO/NiO-350 electrode showed remarkable electrochemical performance for supercapacitors (Chen et al., 2024). By contrasting Zn-Co/MgCo_2_O_4_ nanosheets with Co/MgCo_2_O_4_ nanorods, Bogale et al. showed the important influence of electrode shape on electrochemical performance. Zn–Co/MgCo_2_O_4_’s sheet-like shape improves charge transfer, ion diffusion, and electrolyte accessibility. It achieves a capacitance of 1345 F g^−1^ at 2 A g^−1^ with 83.1% capacitance retention after 10,000 cycles, as opposed to 1224 F g^−1^ for the rod-like structure (Bogale et al., 2025). This emphasizes the importance of nanostructure design in optimizing electrochemical performance.

Despite the fact that many of the doped ZnO and ZnO/graphene-based systems have been widely explored for energy storage applications, a systematic understanding of how controlled dopant concentration influences electrochemical behavior, particularly in conjunction with the conductive carbon matrices, remains insufficient. In this context, the present study introduces a rational design strategy by integrating controlled Cr doping with reduced graphene oxide (RGO). This approach enhances electrochemical efficiency by enabling efficient redox activity through Cr incorporation within the ZnO lattice which can introduce localized electroactive sites, while RGO network provides a conductive pathway for rapid electron transport thereby improving overall charge storage kinetics. Unlike previous reports, this work establishes a direct correlation between the dopant concentration, structural modification and electrochemical performance leading to the identification of an optimized composition with enhanced capacitive behavior and stability.

In this study, Cr-doped ZnO/RGO nanocomposites were synthesized using chemical co-precipitation method (Molaei et al., 2018) with enhanced electrochemical characteristics making them suitable for energy storage devices. Additionally, in order to determine the most effective Cr doping level, this work also provide concentration-dependent optimization strategy. This straightforward co-precipitation approach offers a more sustainable alternative compared to hydrothermal or sol-gel processes as it requires mild synthetic conditions (Gupta and Hegde, 2024). The first step involved the fabrication of ZnO NPs with the varying concentrations of Cr (Cr_x_-doped ZnO, x = 2–10%). The optimal oxidation and reduction capabilities were demonstrated by the prepared 8% Cr-ZnO material. It was followed by the incorporation of the material with reduced graphene oxide to enhance its features further. The coupling of the Cr-ZnO and the RGO offer the synergistic effect owing to its large surface area, highly conductive networks along with the improved diffusion pathways for electrochemical reactions, enhancing its supercapacitive properties as an electrode material. The material was characterized using the UV-visible, energy-dispersive X-ray, SEM, atomic absorption spectroscopy, ICP-OES, and the X-ray powder diffraction.

## 2 Experimental

### 2.1 Chemicals and reagents

The analytical grade products that were acquired from the Sigma Aldrich included the H_2_SO_4_, K_2_S_2_O_8_, phosphoric acid (H_3_PO_4_), KMnO_4_, Zn(NO_3_)_2_.6H_2_O, hydrogen peroxide (H_2_O_2_), NaOH, chromium nitrate nonahydrate (Cr(NO_3_)_3_.9H_2_O), graphite powder, ethanol, distilled water, sucrose, deionized water, ferricyanide, chitosan binder, and acetone.

### 2.2 Synthesis of zinc oxide NPs

To prepare zinc oxide NPs, the chemical precipitation method was employed. To prepare homogeneous solution, 2.974 g of the Zn(NO_3_)_2_.6H_2_O were added to 100 mL of distilled H_2_O in a beaker. Afterwards, 6 g of sodium hydroxide was dispersed in 200 mL of water to create a solution. To create precipitates, a magnetic stirrer was used to agitate this mixture for 4 hours. Next, the precipitates underwent a 15-min centrifugation at 4,000 rpm. Subsequently they were filtered, and washed with the distilled water, suspended in ethanol for 2 h, and dried at 75 °C for 6 h. After being heated to 500 °C for 2 hours in the furnace, the samples were let to cool to room temperature. A pestle and mortar was used to pulverize the nanoparticles thus obtained.

### 2.3 Synthesis of Cr-doped ZnO NPs

Using the chemical co-precipitation technique, Cr-doped zinc oxide NPs (Cr_x_-doped ZnO x = 2–10) were prepared. Initially, 100 mL of zinc nitrate hexahydrate was dissolved into the distilled water to form homogenous solution of 98% zinc nitrate. 2% chromium salt was then added to this solution together with the 6 g of sodium hydroxide to make the 2% Cr-ZnO NPs. The solution was magnetically stirred for 4 h, and then centrifuged at the 4,000 rpm for 15 min. The precipitates were then allowed to suspend in ethanol for 2 h to eliminate the molecules of trapped water. This was followed by the drying of the white precipitates at 75 °C over the period of 6 h. The samples were placed inside furnace at 500 °C for 2 h, then switched off and allowed to cool at the room temperature. The resulting 2% chromium-doped zinc oxide NPs were formed and ground using the pestle and mortar. The process was repeated with the varying concentrations of chromium nitrate nonahydrate to obtain the different chromium-doped ZnO NPs. Table 1. Shows the doping concentration of the chromium salt.

**TABLE 1:** Concentration of chromium and zinc salt.

Percentage	Chromium salt concentration	Zinc salt concentration
2% Cr-Zn	0.1251 g	2.9154 g
4% Cr-Zn	0.2516 g	2.8550 g
6% Cr-Zn	0.3775 g	2.7950 g
8% Cr-Zn	0.5048 g	2.7361 g
10% Cr-Zn	0.6306 g	2.6760 g

### 2.4 GO synthesis using hummers’ approach

The two phases of the modified Hummers method were used to create the GO (Wang et al., 2016). In the first step, 5 g of graphite powder was added to a solution of 300 mL concentrated H_2_SO_4_, 4.2 g K_2_S_2_O_8_ and 6.2 g, and P_2_O_5_. The solution was stirred for the 3 hours, and heated to 80 °C until a dark blue color. After cooling to room temperature, the mixture was diluted using distilled water. To get pre-oxidized graphite, the mixture’s moisture content was eliminated overnight. The second phase included adding 1 g of pre-oxidized graphite to 200 mL of concentrated H_2_SO_4_ that had been refrigerated in an ice bath. 15 g of KMnO_4_ were gradually added to the mixture while it was being violently stirred. After stirring the mixture for 2 hours at 35 °C in a water bath, 3 mL of 30% H_2_O_2_ was gradually added, giving the liquid a vivid yellow hue. To get rid of the metal ion and any remaining acids, the mixture was filtered and repeatedly cleaned with 10% HCl and distilled water. After centrifuging the GO suspension for 40 min at 8,000 rpm, the washing procedure was repeated until the supernatants had a pH of neutral. Finally, the filtered graphene oxide was dried in the hot air oven at 70 °C temperature and the resulting powder was collected for the further use.

### 2.5 Synthesis of reduced graphene oxide

The hydrothermal process was used to synthesize the reduced graphene oxide RGO. First, 100 mg of graphene oxide and 100 mL of deionized water were combined. To guarantee complete mixing, the solution was agitated and kept in an ultrasonic bath for half an hour. The reducing agent, 1 mL of hydrazine hydrate, was then added. The mixture was then moved to a sealed container and cooked for a whole day at 100 °C. The final RGO powder was obtained by centrifuging the black precipitates, washing them many times with distilled water to get rid of any leftovers, and drying them in an oven.

### 2.6 Synthesis of Cr-doped ZnO/RGO nanocomposites

**FIGURE 1:**
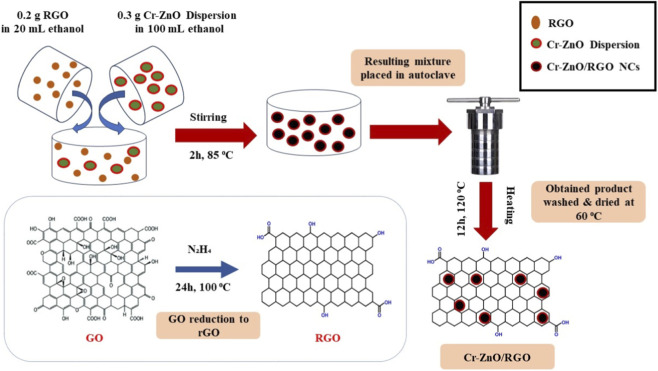
Schematic illustration of the synthesis of Cr-doped ZnO/RGO nanocomposite.

0.3 g of chromium-doped zinc oxide NPs were dispersed in 100 mL of the ethanol. Separately, 20 mL of ethanol was ultrasonically mixed with 0.2 g of the produced reduced graphene oxide for 30 min. Both the solutions were combined and the pH was adjusted using the dropwise addition of sodium hydroxide to facilitate surface activation (Figure 1). The resulting suspension was stirred for 2 h at 85 °C. The mixtures were then placed in a Teflon-lined autoclave and heated to 120 °C for 12 hours. The resulting precipitates were washed with ethanol and dried at 60 °C to obtain the Cr-doped ZnO/RGO nanocomposites.

### 2.7 Electrochemical applications

A high-performance Gamry 3,000 potentiostat in a typical three-electrode configuration was used to assess the electrochemical performance of the produced composite. An Ag/AgCl reference electrode, a platinum wire counter electrode, and a 1M KOH solution were used as the electrolyte. The working electrode was prepared by dispersing 5 mg of the active material in 1.5 mL of distilled water followed by the vortex mixing and ultrasonication for 2 h to obtain homogenous slurry. To improve the adhesion of the active material a 0.5 wt% chitosan-binder solution was prepared separately. The slurry was then drop-cast onto the copper-coated glass substrate (1 cm^2^). The electrode was then dried in an oven using stepwise heating protocol. Initially heated at 60 °C for 10 min followed by the incremental increase of 10 °C every 10 min up to 120 °C in order to ensure the complete solvent evaporation and the stable film formation.

The electrochemical activity of the synthesized samples was assessed through EIS and CV. EIS was used to assess the electrode’s resistive properties throughout a frequency range of 10 kHz to 0.1 Hz. To examine the charge storage behavior, CV measurements were carried out using a potential window of −0.2–0.8 V at different scan speeds (50 to mVs^-1^). GCD measurements were conducted at a constant current density of 1 A g^-1^ within the same potential window in order to further assess the capacitive performance and the cyclic stability. The cyclic stability of the electrode was assessed over the 5,000 charge-discharge cycle, while the capacitive performance was evaluated based on the capacitance retention and Coulombic efficiency.

## 3 Results and discussion

### 3.1 XRD analysis

**FIGURE 2:**
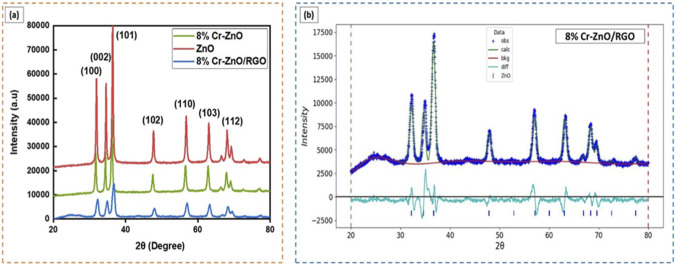
XRD analysis of pure ZnO NPs, 8% Cr-ZnO, and 8% Cr-ZnO/RGO **(a)**. Rietveld refinement of 8% Cr-ZnO/RGO **(b)**.

Figure 2 displays the prepared samples’ XRD pattern. This examination reveals the structural and phase details of the synthesized NPs. The crystal planes (100), (002), (101), (102), (110), (103), (200), and (112) for pure ZnO NPs corresponds to the hexagonal wurtzite ZnO structure, consistent with the JCPDS card number 36-1451 (Ahamed and Kumar, 2016). In the Cr-doped ZnO sample, the diffraction became sharper and intense, indicating improvement in crystallinity due to Cr incorporation. The absence of impurity peaks validates that Cr^3+^ ions were successfully substituted to the Zn^2+^ lattice sites without formation of secondary phases. In the 8% Cr-ZnO/RGO nanocomposite, more intense diffraction peak observed at 24.5 ° corresponding to the (002) plane is indicative of the reduced graphene oxide. This suggest the successful integration of RGO into the ZnO matrix. The overall diffraction pattern remained preserved and consistent with the hexagonal ZnO structure, suggesting that the integration of RGO did not disrupted the ZnO crystalline phase.

Furthermore due to the structural consistency observed for the optimized system, the Rietveld refinement of the 8% Cr-ZnO/RGO nanocomposite was additionally performed via GSAS-II using hexagonal ZnO structural model (space group P6_3_mc) to confirm lattice parameters and phase purity. The refined pattern with R_wp_ value of 8.21% show good agreement between experimental and calculated profiles as shown in Figure 2b. The refined lattice parameters were the a = b = 3.24 Å and c = 5.22 Å which are close to those of the hexagonal ZnO, with the slight variations that can be attributed to the lattice distortions caused by incorporations of the Cr^3+^ ions, confirming high phase purity of material (Yılmaz et al., 2011). The observed changes in the lattice parameter are attributable to the content of foreign atoms and associated lattice defects, as well as the differences in ionic radii between the substituted ions; more particularly, Cr^3+^ ions (0.63 Å) have smaller radius than the Zn^2+^ ions (0.74 Å), and Cr-O bond in the Cr_2_O_3_ are shorter than the Zn-O bond in the ZnO (1.96 Å versus 1.98 Å) (Theyvaraju and Muthukumaran, 2017).

### 3.2 FTIR analysis

**FIGURE 3:**
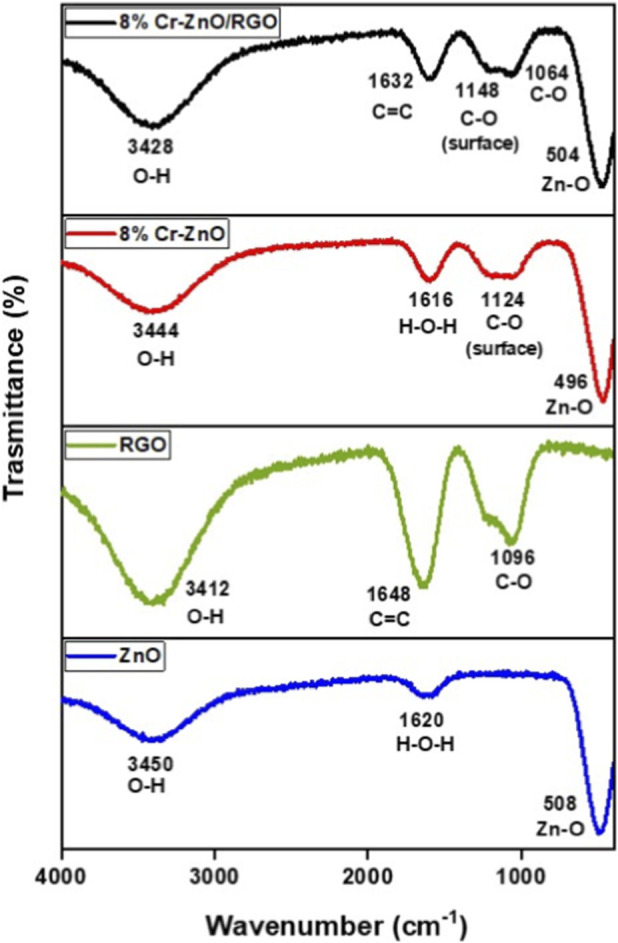
FTIR spectra of ZnO NPs, RGO, 8% Cr-ZnO, 8% Cr-ZnO/RGO.s.

Figure 3 displays the FT-IR spectra of pure ZnO NPs, RGO, 8% Cr-ZnO, and 8% Cr-ZnO/RGO. All of the samples have peaks in the 3,400–3,450 cm^-1^ region, which are ascribed to the -OH stretching vibrations and indicate the presence of either surface-bound moisture or adsorbed water molecules (Rizwana et al., 2020). The peaks in the low frequency region in the range of 400–510 cm^-1^ are indicative of the characteristic ZnO stretching vibrations (Vats et al., 2023). In the spectra of 8% Cr-ZnO, and 8% Cr-ZnO/RGO the band in the range of 1124–1148 cm^-1^ is endorsed to the C-O stretching vibrations associated with surface functional groups originating from the residual species during the synthesis process (Motelica et al., 2022). The presence of this feature in both the samples indicates that it arises from surface species rather than lattice doping. The peaks at 1616 cm^-1^ and 1620 cm^-1^ in the FTIR spectra of 8% Cr-ZnO, and ZnO, respectively, indicates H-O-H bending vibrations (Boukaoud et al., 2021; Hanifah et al., 2015). Similarly, in the spectra of RGO, the peak at 1648 cm^-1^ affirm the partial oxidation of the C=C (Liu et al., 2022). The peaks at 1096 cm^-1^ relate to the C-O extending vibrations in RGO (Ma et al., 2020). In 8% Cr-ZnO/RGO the peaks for C=C and C-O were located at 1632 cm^-1^ and 1064 cm^-1^, respectively. From the findings the retention of the characteristic vibrational peaks were observed in the final composite material. Slight variations in the peaks position suggests minor changes which collectively validates effective interaction between individual components and the successful incorporation within the nanocomposite.

### 3.3 UV-visible spectroscopy

**FIGURE 4:**
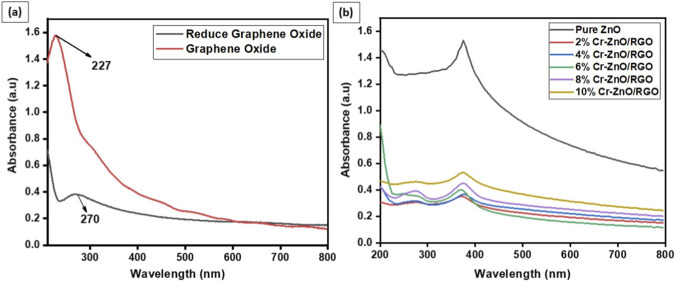
UV-visible spectra of **(a)** GO, RGO, **(b)** Pure ZnO, and (2%–10%) Cr-doped ZnO/RGO.

**FIGURE 5:**
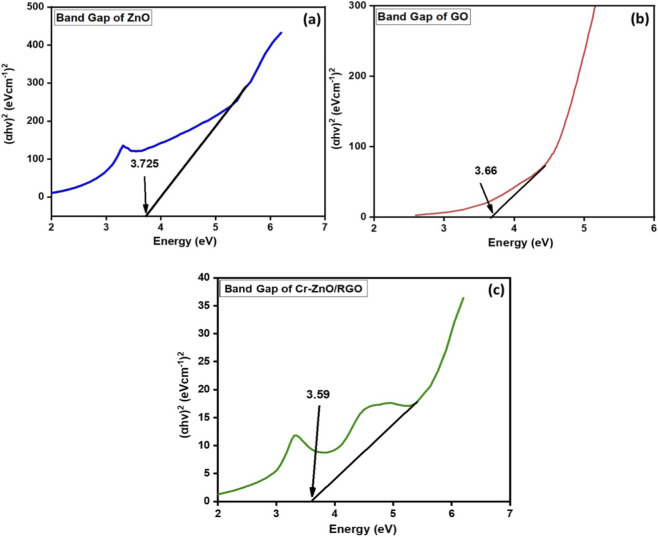
Band gap of Pure ZnO **(a)**, GO **(b)**, and 8% Cr-ZnO/RGO **(c)**.

UV-visible Spectra of graphene oxide, RGO, pure ZnO, and Cr_x_-ZnO/RGO samples are shown in Figure 4. There are prominent absorption peaks at the 270 nm and 227 nm for GO and RGO, respectively, as indicated in Figure 4a. The higher absorption peak in reduced graphene oxide is the result of re-established aromatic conjugation of graphene sheets after the reduction process, confirming the conversion of GO into the RGO. The redshift from the pure ZnO in chromium-doped ZnO/RGO samples give information about the maximum absorption for all of the samples at 355 nm (Figure 4b). The optical band gap (E_g_) was calculated by plotting energy onto the X-axis and (ahv)^2^(eV cm^-1^)^2^ on the Y-axis assuming a direct allowed transition. The band gaps were extracted by extrapolating the linear region of plot to photon energy axis intercept. As seen in Figure 5, the band gaps of pure ZnO is observed to be 3.725 eV, which is higher than the typical bulk value (∼3.2–3.3 eV). This increase can be ascribed to the nanoscale-induced quantum confinement effects, where the restricted motion of the charge carriers can lead to widening of band gap. This behavior has been extensively documented in the literature, where the band gap of ZnO nanostructures can rise dramatically and even reach as high as 3.84 eV. For ZnO nanoparticles made using the chemical precipitation technique, defects like oxygen vacancies and zinc interstitials were also attributed to quantum confinement effects (Samanta, 2018). Recently Goudarzi et al. demonstrated bandgap enlargements as high as 4.2–4.4 eV for the ZnO nanoparticles by employing the co-precipitation method (Goudarzi et al., 2022). Lin et al. prepared ZnO quantum dots with a band gap enlargement of 3.43–3.65 eV using a sol-gel method, allowing for tailoring of their average size through controlled Zn precursor concentration. The size-dependence of UV photoluminescence and absorption spectra evidences the quantum confinement effect (Lin et al., 2005). According to Samanta, as growth time rises, crystallite sizes decrease and the band gap of produced ZnO nanostructures ranges between 3.34 and 3.57 eV. The band gap may be adjusted thanks to this connection. Using a weak quantum confinement model for the emission peaks, the ZnO nanostructures’ visible photoluminescence is ascribed to deep-level transitions (Samanta, 2020). Sajid et al. observed bandgap value of 3.50 for ZnO nanopowder which indicate a blueshift from the 3.37 eV of the bulk ZnO (Ansari et al., 2012).

For GO, and the optimized composition (8% Cr-ZnO/RGO) the band gaps were found to be 3.66, and 3.59 eV, respectively. When the concentration of chromium increases, α-absorption start shifting slightly toward the lower energy (Priscilla et al., 2020). The band gap narrowing in 8% Cr-ZnO/RGO is attributed to the sp-d exchange interactions along with the lattice distortions introduced by Cr^3+^ doping (Qiao, 2018), as well as the synergistic effect of RGO enhancing charge transfer.

### 3.4 Quantitative compositional analysis

The compositional analysis was carried out using the complementary techniques, where, Cr concentration was quantified by AAS due to its high sensitivity for the trace-level dopants. While Zn content was determined by ICP-OES which is well-suited for high-concentration matrix elements with a dynamic range (Planeta et al., 2021). The Zn quantification was not treated as an independent parameter rather it was used to establish the host lattice baseline, enabling the normalization of Cr concentration to determine the actual doping level. This approach ensures that the reported compositions reflect the true incorporation of Cr into the ZnO matrix rather than just relying on the nominal precursor ratios. Oxygen content was not directly measured as ICP-OES and AAS techniques are optimized for metallic elements. Instead oxygen content is inferred based on the stoichiometric ZnO lattice and the validated material composition which is widely accepted for metal oxide systems.

#### 3.4.1 ICP-OES analysis

Elemental analysis was performed using an Agilent 720-ES ICP-OES spectrometer under optimized operating conditions including radio frequency (RF) power of 1.30 kW and argon plasma with an auxiliary flow rate of 1.50 L/min. The measurements were performed at a characteristic Zn emission wavelength in order to ensure reliable quantification. The ICP-OES results present in Table 2 confirm the presence of Zn as the primary matrix element in all samples.

**TABLE 2:** Quantitative elemental composition of Cr-doped-ZnO samples determined by ICP-OES (Zn) and AAS (Cr) and the calculated Cr/Zn ratio.

Sample ID	Zn %	Cr %	Cr/Zn ratio
2% Cr doped ZnO NPs	98.75	2.1	0.021
4% Cr doped ZnO NPs	96.02	4.1	0.043
6% Cr doped ZnO NPs	94.80	6.09	0.064
8% Cr doped ZnO NPs	92.50	8.1	0.088
10% Cr doped ZnO NPs	90.55%	9.93%	0.110

#### 3.4.2 AAS analysis

**FIGURE 6:**
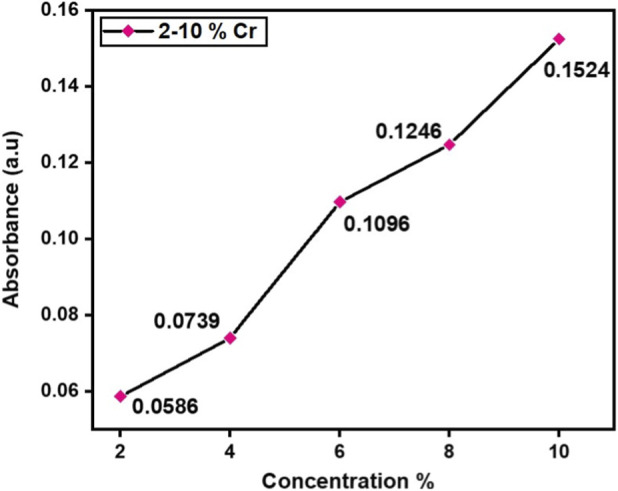
Chromium doping analysis by atomic absorption spectrometer.

The atomic absorption spectroscopy (PerkinElmer Analyst 400) was used to determine chromium doping concentration under the optimized conditions including an air-acetylene flame with a flow rate of 4 L/min and a spectral bandwidth of 0.7 nm at the characteristic Cr wavelength. A five-point linear calibration curve was constructed by plotting absorbance against concentration, yielding a linear relationship with a correlation coefficient (*R* ^2^) calculated to be 0.995, indicating high measurement reliability (Figure 6). The Cr concentration obtained for the doped samples (2%, 4%, 6%, 8%, and 10%) closely matched the nominal values, confirming the controlled and efficient integration of Cr into the ZnO matrix. The systematic increase in Cr concentration across the series further validates the consistency of the synthesis approach and supports the composition trend summarized in Table 2.

The quantitative compositional analysis confirms that the experimentally determined Cr concentrations closely match the nominal doping levels, indicating the efficient incorporation of Cr into the ZnO lattice. A gradual decrease in Zn content with the increasing Cr concentration further supports substitutional doping. The calculated Cr/Zn ratio provides a more reliable representation of the actual doping level and demonstrates a consistent trend across the series.

### 3.5 SEM analysis

**FIGURE 7:**
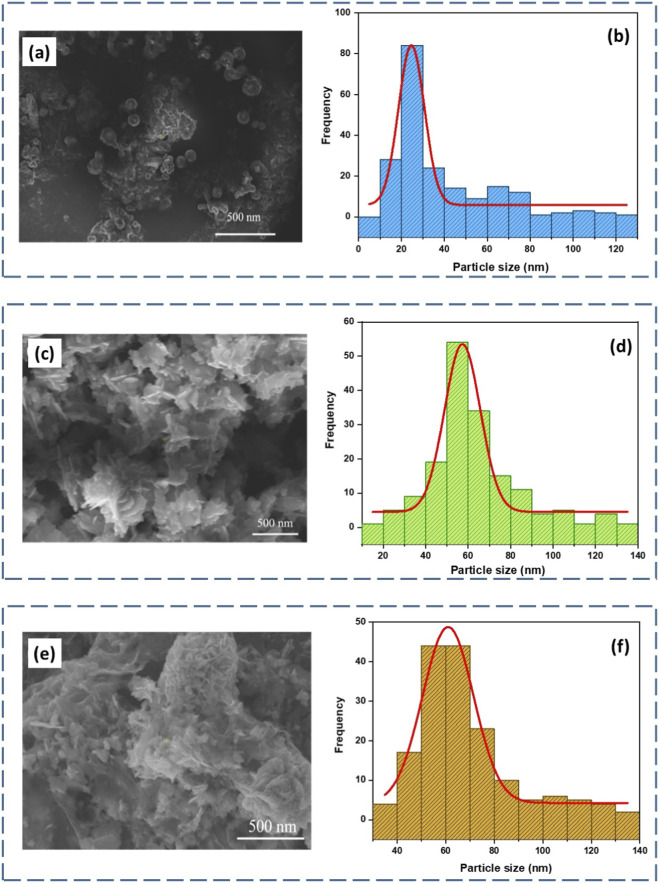
SEM images and corresponding size distribution histograms of **(a,b)** GO, **(c,d)** RGO, **(e,f)** 8% Cr-ZnO/RGO.

The surface morphology of 8% Cr-ZnO/RGO, GO, and RGO was confirmed by the SEM (Figure 7). Graphene oxide SEM image (Figure 7a) reveals loosely stacked thin and wrinkled sheet-like structure which is characteristic of highly oxidized graphene layers (Fusco et al., 2020). The corresponding size distribution histogram shows an average size of 24.7 nm (Figure 7b). Reduced graphene oxide after reduction exhibited more compact, wrinkled, and aggregated morphology, as shown in Figure 7c. This structural change is attributed to the partial removal of the oxygen-containing functional groups leading to the restacking of the graphene layers (Bashir et al., 2025). The size distribution indicates an increased average size of 57.2 nm, consistent with the observed aggregation behavior (Figure 7d). The SEM image of 8% Cr-ZnO/RGO is displayed in Figure 7e. The SEM morphology revealed that the Cr-ZnO nanoparticles appear to be homogenously distributed over the wrinkled surface of the RGO sheets, indicating good interfacial contact between the components. The corresponding size distribution (Figure 7f) further confirms the formation of the composite structure with a slight increase in size to 60.9 nm. The rough, flaky wrinkled architecture of the 8% Cr-ZnO/RGO nanocomposite enhances surface area and connectivity, which are favorable for efficient charge transfer and improve electrochemical activity, indicating potential uses in supercapacitors.

### 3.6 EDX analysis

**FIGURE 8:**
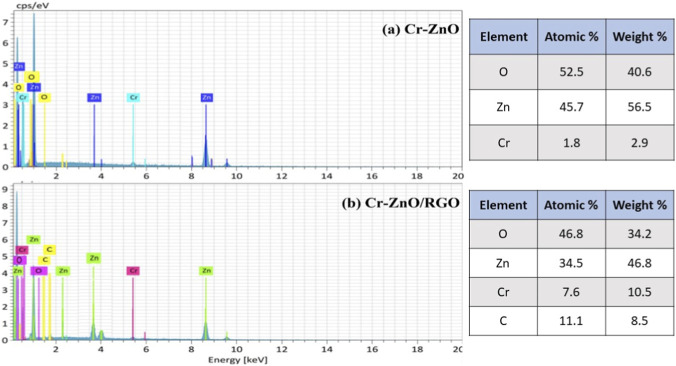
EDX Analysis of **(a)** 8% Cr-ZnO, **(b)** 8% Cr-ZnO/RGO with corresponding quantitative elemental composition.

EDX research reveals details about the synthetic material’s elemental makeup (Qayyum et al., 2024). Figure 8 shows the EDX spectra of fabricated nanomaterials. The presence of Cr, Zn, and O peaks in (Figure 8a) confirms the synthesis of Cr doped ZnO NPs, while the additional peaks of the Carbon (C) and Oxygen in Figure 8b confirm the synthesis of Cr- ZnO/RGO. The corresponding elemental compositions obtained from the EDX analysis is presented alongside the spectra, confirming the existence of Zn, Cr, and O (and C in the composite) which supports the successful dopant incorporation and RGO integration.

### 3.7 Cyclic voltammetry

**FIGURE 9:**
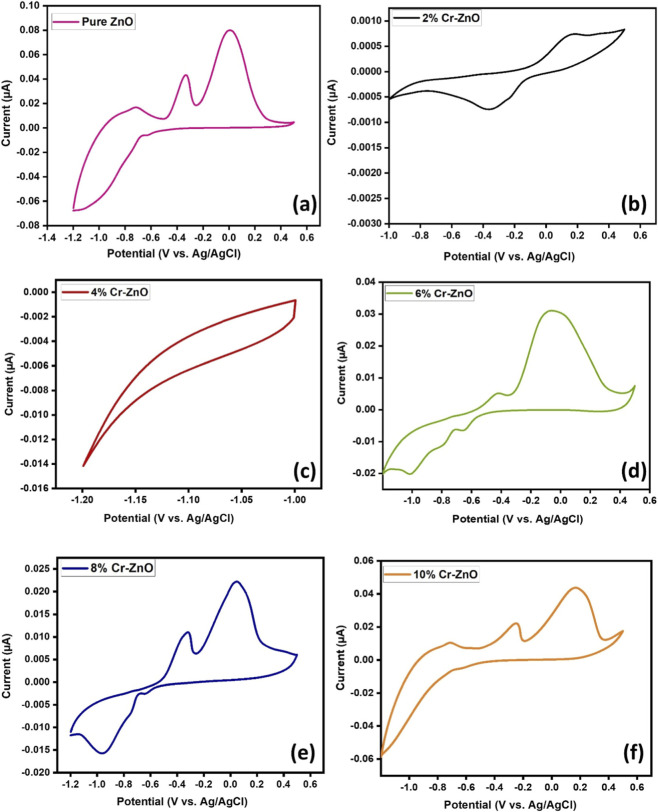
Cyclic voltammetry graphs of Pure ZnO **(a)**, 2% Cr‐ZnO **(b)**, 4% Cr‐ZnO **(c)**, 6% Cr‐ZnO **(d)**, 8% Cr‐ZnO **(e)**, and 10 % Cr‐ZnO **(f)** in 1M KOH at 100mVs‐1 scan rate.

CV analysis was used to assess the electrochemical performance of pure ZnO, 2%–10% Cr-ZnO, GO, RGO, and 8% Cr-ZnO/RGO in 1M KOH. The potential range of −0.2–0.8 V was measured using cyclic voltammetry at various scan speeds between 50 and 100 mVs^-1^.The results for 2%–10% Cr-ZnO at 100 mV/s are established in Figure 9, as they exhibited clear electrochemical responses and minimal distortion at this scan rate. The CV curve of pure ZnO showed a pronounced oxidation peak and small reduction peak (Figure 9a). 2% Cr-ZnO showed a small oxidation peak and a large reduction peak (Figure 9b), while 4% Cr-ZnO did not showed distinct oxidation-reduction peak even at a low scan rate (Figure 9c). 6% Cr-ZnO showed good oxidation peak but an imperfect reduction peak (Figure 9d). The CV curve of 8% Cr-ZnO displayed both oxidation and reduction peaks, forming a duck-shaped curve observed during the cyclic potential scan (Figure 9e). This improved response can be attributed to that of the optimal Cr-doping level which might favorably influence the charge transport and the electrochemical kinetics. In contrast, the CV profile of 10% Cr-ZnO (Figure 9f) exhibits a discernible redox pair, however the increased distortion peak and separation suggests high polarization.

The produced electrodes’ energy storage technique combines Faradic pseudocapacitance with EDLC. The redox peaks seen in the CV curves are indicative of reversible Faradic reactions that take place at the electrode-electrolyte interface linked to the metal oxide’s surface or near-surface electroactive sites and its interaction with the electrolyte, which are further influenced by the inclusion of Cr (Ramachandran et al., 2022). The variation in redox peak features across different compositions offer insights into the electrochemical behavior of the system. Materials exhibiting a single broad peak typically indicate overlapping or kinetically hindered redox processes, where oxidation and reduction reactions are not well-resolved due to limited conductivity or fewer accessible active sites. In contrast, the presence of a pair of well-defined redox peaks corresponds to the distinct and reversible oxidation-reduction reactions, indicating improved electrochemical kinetics and better charge transfer behavior (Kalaivani et al., 2026). Among the investigated compositions, the 8% Cr-ZnO offers an optimal balance of electroactive sites, whereas lower doping results in insufficient active centers and higher doping leads to increased polarization and reduced electrochemical reversibility.

**FIGURE 10:**
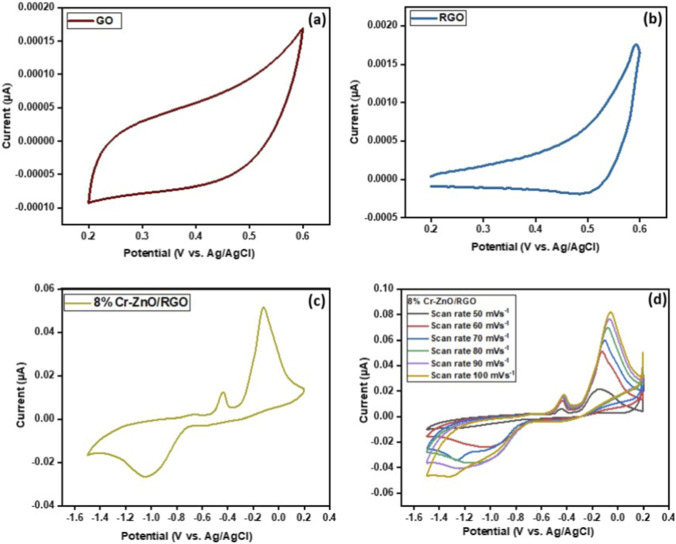
CV graphs of **(a)** GO, **(b)** RGO, **(c)** 8% Cr-ZnO/RGO nanocomposite, and **(d)** 8% Cr-ZnO/RGO nanocomposite at different scan rates (50–100 mVs^-1^).

GO displayed a quasi-rectangular shape at a 100 mVs^-1^ scan rate, suggesting good capacitive properties, as shown in Figure 10a. The same behavior was observed in reduced graphene oxide (Figure 10b). Figure 10c shows the electrochemical activity of 8% Cr-ZnO/RGO at the scan rates that ranging from 50 to 100 mVs^-1^. The 8% Cr-ZnO/RGO electrode exhibited the most prominent redox activity and highest specific current response at 100 mVs^-1^, indicating significant charge storage capacity. Upon the integration of RGO, the electrochemical performance is significantly enhanced due to the formation of a conductive network that facilitates rapid electron transport and improves the utilization of electroactive sites. The synergistic interaction results in more defined and reversible redox peaks, reduced polarization and improved charge transfer kinetics in the 8% Cr-ZnO/RGO compared to the pristine and Cr doped ZnO samples. The improved behavior confirms that the composite system effectively combines enhanced electrical conductivity with optimized redox activity, leading to superior electrochemical performance (Figure 10d). Even though the presence of redox peaks in the CV curves indicates the contribution of Faradic responses, the overall quasi-rectangular shape and good rate response suggest that surface–controlled capacitive behavior remains dominant, with partial diffusion-controlled contributions. This confirms that the electrode exhibits pseudocapacitive characteristics rather than purely battery-type behavior.

The specific capacitance (Csp) values were obtained using the equation below, and are listed in Table 3.

$$C_{\text{sp}}=\frac{\int Idv}{S\Delta Vm}$$

ΔV , ∫Idv,S,m refer to the integrated area of the CV curve scan rate, mVs^-1^, mass of active surface area.

**TABLE 3:** Specific capacitance (C_sp_) of Pure ZnO, GO, RGO, 2%–10% Cr-ZnO, 8% Cr-ZnO/RGO.

Material	*C*_*sp*_ *(Fg* ^*-1*^ *)*
Pure ZnO	154.68
GO	1.16
RGO	5.71
2% Cr-ZnO	14.57
4% Cr-ZnO	11.88
6% Cr-ZnO	54.87
8% Cr-ZnO	390.78
10% Cr-ZnO	91.62
8% Cr-ZnO/RGO	703.89

### 3.8 Electrochemical impedance spectroscopy (EIS)

**FIGURE 11:**
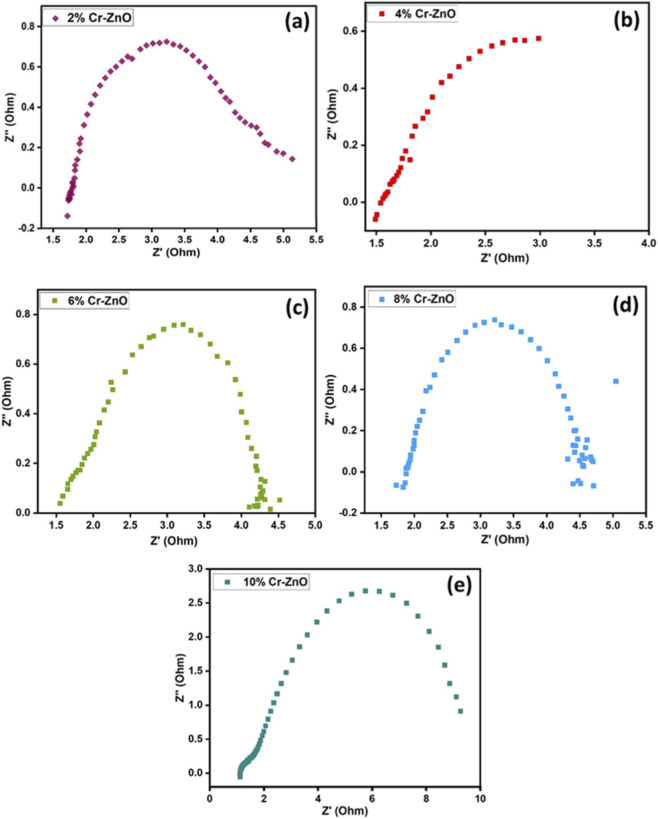
Nyquist Plots of 2% Cr‐ZnO **(a)**, 4% Cr‐ZnO **(b)**, 6% Cr‐ZnO **(c)**, 8% Cr‐ZnO **(d)**, and 10 % Cr‐ZnO **(e)**.

**FIGURE 12:**
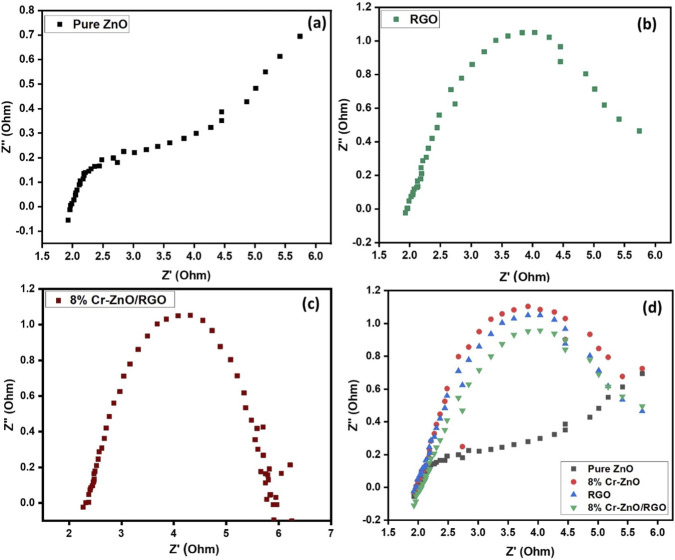
Nyquist plots of **(a)** pure zinc oxide, **(b)** RGO, **(c)** 8% Cr-ZnO/RGO433333333333g433333333333g3nj, and comparative analysis **(d)**.

EIS was employed to analyze the conductive properties, electron transport characteristics, and surface-induced processes of the synthesized electrodes (Li et al., 2022). These tests were accomplished in a H_2_S saturated environment over a wide frequency range, at an AC voltage of 5V and a DC bias of 0.8V to generate Nyquist plots. Figure 11 illustrates the comparison of the Cr_x_-ZnO (x = 2–10%) NPs, while Figure 12 depict the EIS spectra of pure ZnO, RGO, and 8% Cr-ZnO/RGO electrode. Significantly, the Nyquist plot for the 8% Cr-ZnO/RGO shows a straight line in the low-frequency area and a semicircle in the high-frequency region, suggesting improved diffusion processes and charge transfer resistance (Figure 12c) (Zhao et al., 2022).

When compared to the other produced samples, the 8% Cr-ZnO/RGO nanocomposite’s semicircle in the Nyquist plot has a smaller diameter, which indicates excellent charge transfer kinetics (Figure 12d). The values of surface resistance (Ru) and solution resistance (Rp) derived after fitting the Nyquist data with the Randles equivalent circuit are summarized in Table 4. Compared to all the samples, the nanocomposite showed considerably lower Ru and Rp values indicating enhancement in the efficiency of charge transfer. This enhancement can be explained by the synergistic behavior of Cr, ZnO and RGO in the composite. On the whole, the EIS analysis implies that the integration of RGO into the Cr-ZnO matrix significantly increased the conductivity and the electrochemical performance of the electrode.

**TABLE 4:** Calculated Ru and Rp values of Pure ZnO, 2%–10% Cr-ZnO, RGO, and 8% Cr-ZnO/RGO.

Material	Ru resistance (Ohm)	Rp resistance (Ohm)
Pure ZnO	5.2	20.6
2%Cr-ZnO	2.924	2.585
4%Cr-ZnO	2.1707	3.130
6%Cr-ZnO	2.001	2.738
8%Cr-ZnO	1.722	1.290
10%Cr-ZnO	1.852	2.841
RGO	2.195	2.3443
8%Cr-ZnO/RGO	1.168	0.957

### 3.9 GCD and cyclic stability analysis

**FIGURE 13:**
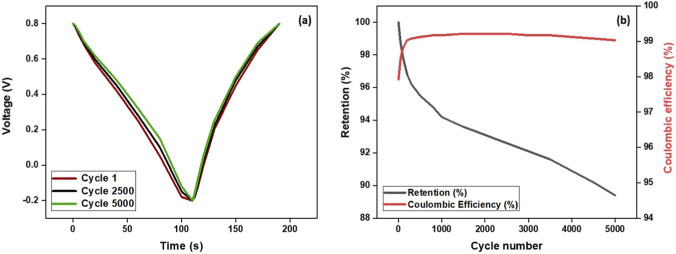
GCD profiles of the optimized 8% Cr‐ZnO/RGO electrode at different cycling stages (1st, 2500th, 5000th) **(a)**. Cycling stability of the electrode over 5000 charge-discharge cycles demonstrating capacitance retention and coulombic efficiency **(b)**.

GCD tests were used to further assess the electrochemical accomplishment of the electrode material. Figure 13 displays the GCD profiles of the 8%Cr-ZnO/RGO nanocomposite which exhibit quasi-symmetric charge-discharge characteristics with slight deviations from the ideal linearity. This is consistent with the pseudocapacitive behavior observed in the CV analysis. A small IR drop is observed which is in agreement with the low internal resistance obtained from the EIS measurements. The representative GCD curves at the different cycling stages (1^st^, 2500th, 5000th) show nearly overlapping time-voltage profiles with minimal distortions (Figure 13a), confirming good reversibility and structural stability during the prolonged cycling. The cyclic performance demonstrates that the electrode retains approximately 89% of its initial capacitance after 5,000 cycles (Figure 13b). In addition, the Coulombic efficiency remain close to ∼99% throughout the cycling process, indicating highly reversible charge-discharge behavior with minimal energy loss. The combined contribution of capacitive and Faradic redox responses suggests a hybrid charge storage mechanism which is beneficial for achieving stable and efficient electrochemical performance in aqueous supercapacitor systems (Ram et al., 2023).

## 4 Limitations and future outlook

This work successfully illustrates the improved electrochemical activity of Cr-ZnO/RGO nanocomposite and its potential applicability in supercapacitor technology. The gradual increase in capacitance, especially at optimal doping rate reveals the possibility of the composite material to use conveniently in energy storage applications. Moreover, charge transport was greatly enhanced when reduced graphene oxide was incorporated as the conductive substrate which ensured the positive synergistic effects of the composite material. The assessment was, however, confined to the three-electrode set-up with 1M KOH aqueous electrolyte. Although such arrangement is useful for material level analysis, it might not be representative of the behavior of the material in an actual device configuration. Future experiments may consider two-electrode systems, both of the symmetric and asymmetric configurations, to be more stimulating of real-world applications. Besides, investigating broader potential window and evaluating long-term mechanical and electrochemical stability under realistic operating circumstances is also suggested. This will assist in the development of scalable supercapacitor technologies out of promising lab-scale results.

## 5 Conclusion

This work employed a straightforward procedure to synthesize Cr doped ZnO and Cr-ZNO/RGO nanomaterials for electrochemical energy storage applications. Electrochemical analysis including CV and EIS revealed that 8% Cr-doped ZnO presented a high specific capacitance (390 F g^-1^) compared to other doping levels, highlighting the significance of the optimal dopant concentration. Upon the integration of RGO, the capacitance is further increased to 703 F g^-1^, indicating improved electrochemical performance due to synergistic effects between the individual components within the composite matrix. EIS analysis revealed reduced resistance (Ru = 1.168 Ω, Rp = 0.957 Ω), confirming improved charge transport properties in the 8% Cr-ZnO/RGO. Additionally, GCD measurements demonstrate good cyclic stability, with ∼89% capacitance retention and ∼99% Coulombic efficiency after prolonged cycling. These results suggest that using conductive carbon structures in conjunction with controlled Cr doping might be a potential strategy for creating high-performance electrode materials. A helpful foundation for the development of cutting-edge electrode materials for energy storage applications is provided by this research.

## Data Availability

The original contributions presented in the study are included in the article/supplementary material, further inquiries can be directed to the corresponding authors.
